# Expression and Clinical Significance of Decoy Receptor 3 in Acute-on-Chronic Liver Failure

**DOI:** 10.1155/2019/9145736

**Published:** 2019-06-18

**Authors:** Su Lin, Bing Wu, Yehong Lin, Mingfang Wang, Yueyong Zhu, Jiaji Jiang, Lurong Zhang, Jianhua Lin

**Affiliations:** ^1^Liver Center, The First Affiliated Hospital of Fujian Medical University, Fuzhou, Fujian 350005, China; ^2^Fujian Key Lab of Individualized Active Immunotherapy and Key Lab of Radiation Biology of Fujian Province Universities, Fuzhou 350005, China; ^3^Department of Radiation Oncology, College of Medicine, University of Florida, Gainesville, Florida 32610, USA; ^4^Lab of Radiation Biology, Fujian Provincial Tumor Hospital, Fuzhou 350006, China

## Abstract

**Aims:**

To explore the expression level and clinical significance of decoy receptor 3 (DcR3) in patients with acute-on-chronic liver failure (ACLF).

**Methods:**

Serum DcR3 levels were measured by enzyme-linked immunosorbent assay (ELISA) in 76 patients with ACLF and 41 non-ACLF patients with chronic liver disease. Blood routine and liver functions were accessed for their correlations with DcR3.

**Results:**

Serum DcR3 in ACLF patients was significantly higher than that in non-ACLF patients. It was positively correlated with neutrophilic granulocyte, aspartate aminotransferase, prothrombin time, and international standardized ratio, but negatively correlated with platelet and serum albumin. At the early stage, the level of DcR3 was not significantly different between the survival and nonsurvival group of ACLF. However, at the late stage, DcR3 increased in nonsurvival and gradually decreased in survivals. The baseline DcR3 could not sufficiently predict the outcome of ACLF, while the change of DcR3 within the first week displayed a better predictive value than model for end-stage liver disease (MELD) score.

**Conclusions:**

DcR3 was highly expressed in patients with ACLF and correlated with several clinical indices. Dynamic change of DcR3 might predict the prognosis of ACLF.

## 1. Introduction

Acute-on-chronic liver failure (ACLF) is a fetal clinical syndrome featured by rapid development of massive hepatocellular dysfunction, with an extremely high mortality rate around 50% [1–4]. Tumor necrosis factor (TNF) induced apoptosis plays an important role in the development of liver failure [5, 6]. The surge of proinflammatory cytokines in ACLF patients, such as TNF-*α* and interleukin 6 (IL-6), is comparable to those described in sepsis [5, 7, 8].

Decoy receptor 3 (DcR3), a soluble decoy receptor with a binding domain but without a transmembrane domain, belongs to TNF receptor superfamily [9]. It competitively binds and neutralizes Fas ligand (FasL), modulates immune responses, and suppresses TNF-induced apoptosis [10–12]. In various cancers, DcR3 helps cells to evade the host immune surveillance [13, 14]. In inflammatory diseases and autoimmune diseases, DcR3 modulates the differentiation and maturation of immune cells like monocyte, macrophage, and negatively regulates the activation of B cells by Toll like receptor ligands [12, 15, 16]. Recently, DcR3 is recognized as a novel biomarker for sepsis and its serum level is correlated with procalcitonin (PCT) [17–19]. DcR3 has also been shown to protect liver injuries in animal models by suppressing inflammation [20, 21].

However, the usefulness of DcR3 in ACLF has never been investigated. We speculated that the serum level of DcR3 might be altered in ACLF and served as a useful biomarker for ACLF. Therefore, we analyzed serum DcR3 in patients with or without ACLF for its clinical value.

## 2. Materials and Methods

### 2.1. Sample Collection

Sera collected from patients hospitalized with or without ACLF in the First Affiliated Hospital of Fujian Medical University from July 2012 to July 2016 were used. Blood samples from patients with ACLF were continuously collected at 3 days, 7 days, and 14 days after admission. The serum was divided into 0.5 ml/tube and stored at -80°C until analysis. All patients were followed up for 3 months after admission.

The diagnosis of ACLF was made according to the guideline of the Asian Pacific Association for the Study of the Liver (2014): the development of jaundice (total serum bilirubin [TBIL] ≥5mg/dl) and INR≥1.5 or prothrombin activity [PTA] ≤40%) within 4 weeks, complicated with ascites and/or encephalopathy [22].

### 2.2. Data Collection

The following data were collected from all patients: age, sex, etiology of liver disease, PCT, C-reaction protein (CRP), white blood cell (WBC), percentage of neutrophils (N%), and liver and renal function tests on admission (0 day). For patients with liver failure, those indices were also collected at 3, 7, and 14 days after admission.

A quantitative ELISA was used to measure DcR3 as previously reported [23]. The DcR3 standards were run simultaneously in the same assay for the calculation of unknowns. The intra-CVs of assays were <5-10% as defined after testing of 20 wells of same plasma spiked with low, medium, or high concentration of DcR3, respectively, in the same plate. The intra-CVs were calculated as standard deviation/mean value of 20 wells. Similarly, the tests were performed in three different batches of plates; then inter-CVs <8-15% were calculated from the test results of three different batches.

### 2.3. Statistical Analysis

Statistical analyses were performed by the SPSS software, version 18.0 (SPSS, Chicago, IL, USA). The normality of the distribution was estimated by the Kolmogorov-Smirnov test. Continuous variables were represented as mean ± standard deviation or median (interquartile range) [24] and compared using the Student's* t-*test in the case of normal distribution or the Mann-Whitney* U* test in the remaining cases. Categorical variables were expressed as counts (percentages) and evaluated by Chi-squared test or the Fisher's exact test when the number of samples was limited. The correlations of PCT levels with the other indicators were analyzed using the Pearson correlation test and the association between the two variables was measured as Pearson's correlation coefficient(r). The diagnostic accuracy for DcR3 and other indicators for the outcome of ACLF were expressed as the area under the receiver operating characteristic curve (AUROC). A* p* value<0.05 was considered statistically significant.

### 2.4. Ethics

The study was approved by the institutional ethics review board of First Affiliated Hospital of Fujian Medical University (protocol# 2015-084) and was in compliance with the Declaration of Helsinki.

## 3. Results

### 3.1. Characteristics of Patients and Differences in Biomarkers

A total of 117 patients with available blood samples were included in this study, 76 (65.0%) patients with ACLF (ACLF group) and 41(35%) with liver cirrhosis (non-ACLF group). The details of clinical characteristics and laboratory data are shown in Table 1. Approximate 80% of patients were infected by hepatitis B virus (HBV). No significant difference in sex, age, and etiologies of liver diseases was found between these two groups.

ACLF patients had higher TBIL, PCT, alanine aminotransferase (ALT), aspartate aminotransferase (AST), *γ*-glutamyl-transferase (*γ*-GT), WBC, N%, plasma ammonia, prothrombin time (PT), and international normalized ratio (INR) levels than non-ACLF patients, while the albumin and platelet levels were significantly lower in ACLF patients.

The DcR3 level was significantly higher in ACLF patients than in non-ACLF patients [0.97(0.17-2.32) ng/mL versus 0.21(0.11-0.49) ng/mL, p<0.001].

### 3.2. Correlation of DcR3 with Clinical Biomarkers

Pearson correlation test was used to explore the correlations of PCT levels with the other indicators in overall population. The results showed that DcR3 levels were positively correlated with TBIL (r=0.185, p=0.049), N% (r= 0.262, p=0.005), PT (r=0.349, p<0.001), INR (r=0.344, p<0.001), AST (r=0.274, p=0.003), and *γ*-GT (r=0.216, p=0.021) and negatively correlated with platelet (r=-0.196, p=0.035) and albumin (r=-0.273, p=0.003), indicating that the DcR3 levels reflected the severity of liver damage.

### 3.3. DcR3 for the Severity of ACLF

**Figure 1:**
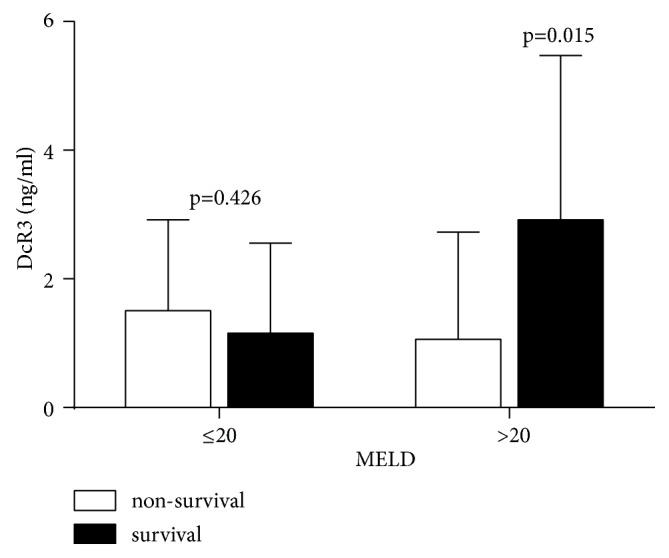
*DcR3 level correlated with MELD score*. Seventy-six patients with ACLF were divided into two groups, nonsurvival or survival group, and, then based on their MELD score, further divided into the score ≥ 20 and <20 groups. The DcR3 levels were compared among these groups. Only in the MELD score ≥20 group, there was the difference of DcR3 statistically significant.

Among 76 patients with ACLF, 38 (50%) died within 3 months. There was no difference in patients' age and sex between the survival group and nonsurvival group. But, the TBIL, N%, PT/INR, and model for end-stage liver disease (MELD) score in nonsurvival group were significantly higher than those in survival group (Table 2). Although the average DcR3 level on admission was slightly lower in nonsurvivals (1.27±1.54 ng/mL) than that in survivals (1.80±2.06 ng/mL), the difference was not statistically significant. However, DcR3 levels were significantly higher in patients with MELD score ≥ 20 than those with the score <20 (2.91 ng/mL versus 1.06 ng/mL, p=0.015, Figure 1), indicating that the DcR3 levels reflect the deteriorating condition of ACLF during the disease progression.

### 3.4. Differences of DcR3 in ACLF Patients with and without Infection

According to previous report, DcR3 level correlated with sepsis [17, 18]. We compared the differences of DcR3 levels in ACLF patients with and without infection. Among the 76 patients with ACLF, 35 patients were diagnosed with bacterial infection, and 41 patients were without. The WBC, N%, and CRP levels were significantly higher in patients with infection, while the DcR3 and PCT levels were comparable between two groups (Table 3).

### 3.5. The Predictive Value of DcR3 for the Outcome of ACLF

**Figure 2:**
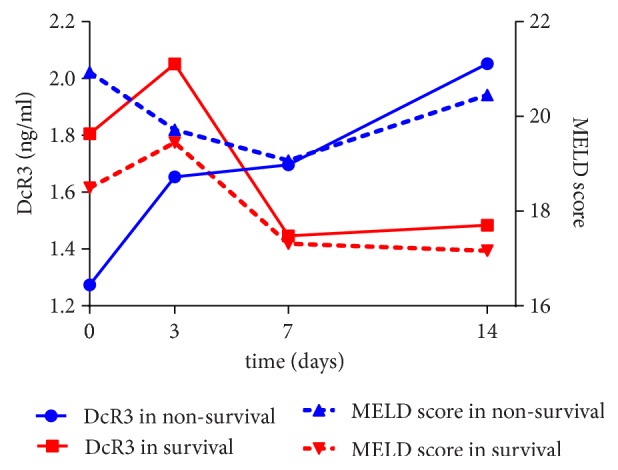
*Dynamic alterations of DcR3 and MELD score*. Of 76 ACLF patients, 43 patients were studied for both serum DcR3 level and MELD score. The DcR3 level on admission was slightly lower in nonsurvival (1.27±1.54 ng/mL) than in survival (1.80±2.06ng/mL); the difference was not statistically significant. However, after 7 days of admission, the patients in nonsurvival group had a steady increase of serum DcR3. The increase was positively correlated with MELD score. The DcR3 decreased along with MELD score in the patients of survival group.

The predictive accuracies of DcR3 and MELD score for 3-month survival in ACLF patients were compared. The AUROC of baseline MELD score (0.645, 95% confident interval (CI): 0.521-0.769, p=0.030) was significantly higher than baseline DcR3 (0.452, 95%CI: 0.320-0.584, p=0.470), indicating a poor predictive value of baseline DcR3. Then we compared the changes of DcR3 and MELD score within the first week in patients who had serial DcR3 data. Of 76 ACLF patients, 43 patients have serial collections of serum samples for DcR3 tests. On the 7 days of admission, the patients in nonsurvival group had a steady increase of serum DcR3 (0.15±0.20 ng/mL), while the DcR3 was declined in the patients of survival group (-0.45±0.21 ng/mL) (Figure 2). The changes of DcR3 between survival and nonsurvival groups were significantly different (p=0.046). However, the changes of MELD score within the first week were comparable between survival and nonsurvival group (-0.93±2.05 versus -1.22±0.72, p=0.897). The difference of DcR3 within the first week displayed a better predictive value (AUROC 0.709, 95%CI: 0.533-0.886, p=0.024 ) than the changes of MELD (0.606, 95%CI: 0.423-0.788, p=0.245) or even the baseline MELD score in patients with serial data (0.519, 95%CI 0.360-0.676, p=0.836).

## 4. Discussion

This study revealed for the first time that (1) serum level of DcR3 in ACLF patients was significantly higher than that of no-ACLF, (2) DcR3 level was positively correlated with TBIL, N%, PT, INR, AST, and *γ*-GT, but negatively correlated with platelet and albumin, (3) DcR3 was not significantly different between the survival and nonsurvival groups of ACLF at the early stage of the disease. However, it steadily increased in nonsurvival group, while it gradually decreased in the survival group, and (4) the steadily increased serum DcR3 level in patients with ACLF indicates a poor outcome.

While the elevated DcR3 in ACLF is not reported previously, an increased DcR3 has been observed in patients with chronic HBV infection and is also suggested to be a useful noninvasive biomarker for discrimination of active hepatitis B from inactive HBV carriers [25] and a marker for liver fibrosis [26]. The pathophysiological value of increased DcR3 might be related with its function of modulating immune response, disrupting Fas signaling, and suppressing apoptosis.

The pathological feature of ACLF is the massive hepatocyte death resulting from an excessive immune response targeting liver cells [27]. This inflammatory response not only is localized in the liver, but also spreads to the whole body system, leading to a systemic inflammatory, similar to sepsis [7, 8, 28]. Excessive and uncontrollable inflammation might further deteriorate liver injury [27]. Previous studies showed that DcR3 was elevated in septic patients and could be a useful prognostic biomarker [17, 29]. In this study, it was shown that the baseline of DcR3 levels was not significantly different between surviving and nonsurviving patients with ACLF. A possible explanation for this might be that, at the first few days of acute damage, the DcR3 responds at a similar magnitude; thus the single point data was not sufficient to reflect the whole picture of the disease. Only the follow-up observation of DcR3 could represent better its clinical value as biomarker for the prognosis of ACLF. As demonstrated in Figure 2, in patients who received multiple DcR3 tests during days 3 to 14, DcR3 levels declined in survival patients, while they continually increased in patients with poor prognosis, indicating that the dynamic observation of DcR3 could better represent ACLF progression and prognosis. The ROC analysis confirmed that the changes of DcR3 in the first week were a better predictive biomarker for the prognosis of ACLF.

In terms of mechanism, DcR3 reduces apoptosis in ACLF by blocking Fas signaling. The elevation of DcR3 in nonsurvived patients might be the response to overactivated Fas signaling. Higher DcR3 levels may indicate a greater magnitude of inflammation and poor outcome. However, endogenous DcR3 might not be enough to overcome the overwhelming effect of proapoptotic factors in ACLF patients with poor outcome. Exogenous DcR3 analog has been shown to attenuate Fas L-induced apoptosis in fulminated liver injury and reduce the death rate [30]. Therefore, treatment targeting Fas-DcR3 pathway might be a promising therapeutic approach for ACLF.

Taken together, DcR3 levels are elevated in ACLF patients. Continuous increase of DcR3 levels might be a sign of poor outcome.

The major weakness of this study was that it was a single-center retrospective study with small-size samples. Multicenter studies with larger samples are needed to address the significance of DcR3 in ACLF and the possible therapeutic potential of DcR3 or its analogs.

## Figures and Tables

**Table 1:** Comparison of clinical characteristics and biomarkers between ACLF and non-ACLF groups of patients.

	ACLF (n=76)	Non-ACLF (n=41)	P value
Age (years)	47.49 ± 15.24	43.95 ± 14.90	0.230
Male (%)*∗*	60 (78.95%)	30 (73.17%)	0.481
TBIL (*μ*mol/L)	311.99 ± 160.46	79.96 ± 98.28	*<0.001*
Albumin (g/L)	30.65 ± 4.73	37.47 ± 6.26	*<0.001*
ALT (U/L)	220(100.75--703.00)	58 (32.00--227.00)	*<0.001*
AST (U/L)	207 (115.00--449.75)	58(30.00--163.00)	*<0.001*
*γ*-GT (U/L)	117(78.25--210.50)	59(27.00--135.00)	*0.001*
WBC (×10^9^/L)	6.31(4.61--8.68)	5.19 (3.77--6.41)	*0.007*
N%	69.80 ± 10.39	57.21 ± 11.43	*<0.001*
Platelet (×10^9^/L)	104.50 (63.00--135.75)	140.5 (98.00--201.50)	*0.002*
CRP(mg/L)	18.54(9.80--18.54)	12.44 (6.33--25.85)	0.333
PCT(ng/mL)	0.76(0.40--1.44)	0.43 (0.21--0.78)	*0.011*
PT(s)	21.05(17.33-24.93)	13.05 (12.25-14.88)	*<0.001*
INR	1.82 (1.49-2.24)	1.12 (1.02-1.29)	*<0.001*
Serum creatinine(umol/L)	63.00 (50.25-70.00)	64.20 (51.63-79.35)	0.273
DcR3(ng/mL)	0.97 (0.17- 2.32)	0.21 (0.11-0.49)	*<0.001*
Etiologies*∗*			0.737
HBV-related	64(82.89%)	33(80.49%)	
others	9(13.16%)	5(12.20%)	
Alcoholic	3(3.95%)	3(7.32%)	

*∗* Data in this table were obtained upon admission and expressed as the number of patients (percentage), mean ± standard deviation, or mean (interquartile range).

**Table 2:** Comparison of clinical characteristics between survived and nonsurvived ACLF patients.

	Non-survivals(n=38)	Survivals(n=38)	P value
Age(years)	50.24 ± 16.72	44.74 ± 13.25	0.116
Male (%)*∗*	28 (73.68%)	32 (84.21%)	0.266
TBIL(*μ*mol/L)	376.71 ± 184.18	247.26 ± 98.30	*<0.001*
Albumin(g/L)	31.03 ± 4.56	30.26 ± 4.92	0.478
ALT (U/L)	202.50(85.00--856.25)	263.00(107.5--327.25)	0.901
AST(U/L)	201.00(128.25--571.75)	252.00(115.00--445.25)	0.954
*γ*-GT (U/L)	111.00(63.25--210.50)	139.00(88.25--213.00)	0.306
WBC(×10^9^/L)	6.65(4.94--9.31)	6.09 (4.15--8.10)	0.187
N%	72.84 ± 8.96	66.77 ± 10.94	*0.010*
Platelet(×10^9^/L)	88.00 (62.00--125.00)	121.00 (67.50--145.00)	0.245
Plasma ammonia (*μ*mol/l)	64.85 ± 34.12	70.51 ± 27.81	0.444
Blood lactic acid	2.77 (2.20--3.50)	2.39 (1.87--3.08)	0.124
PT(s)	23.05(18.50--23.05)	19.8(16.78--23.95)	*0.042*
INR	2.00(1.60--2.44)	1.73(1.45--2.10)	*0.041*
CRP(mg/L)	19.15(10.06--27.64)	17.15(7.28--25.42)	0.421
PCT(ng/mL)	0.67 (0.38--1.24)	0.86 (0.53--1.84)	0.136
Serum creatinine(umol/L)	60.00(49.00--70.50)	63.00(35.00--172.00)	0.571
MELD score	21.37 ± 5.71	18.48 ± 5.11	*0.022*
MELD-Na score	19.38 ± 10.98	14.54 ± 8.92	0.039

*∗*Data in this table were obtained upon admission and expressed as the number of patients (percentage), mean ± standard deviation, or mean (interquartile range).

**Table 3:** Comparison of DcR3 and other biomarkers among ACLF patients with and without bacterial infection.

	Infection (n=35)	None- infection(n=41)	P value
DcR3(ng/mL)	1.64 ± 2.04	1.45 ± 1.65	0.651
PCT(ng/mL)	1.91 ± 3.36	1.01 ± 1.30	0.141
CRP(mg/L)	27.68 ± 28.38	16.25 ± 9.61	*0.036*
WBC(×10^9^/L)	8.26 ± 4.74	6.37 ± 2.45	*0.038*
N%	73.75 ± 9.97	66.43 ± 9.63	*0.022*

## Data Availability

The data are available from the first author (Su Lin) upon reasonable mutual agreements.

## Abbreviations

DcR3: Decoy receptor 3
ACLF: Acute-on-chronic liver failure
MELD score: Model for end-stage liver disease score
TBIL: Total serum bilirubin
INR: International normalized ratio
PTA: Prothrombin activity
ALB: Albumin
ALT: Alanine aminotransferase
AST: Aspartate aminotransferase
*γ*-GT: *γ*-glutamyl-transferase
WBC: White blood cells
N%: Percentage of neutrophils
PT: Prothrombin time
PA: Plasma ammonia
PCT: Procalcitonin
CRP: C-reaction protein
IL-6: Interleukin 6.
